# Epizootics due to Yellow Fever Virus in São Paulo State, Brazil: viral dissemination to new areas (2016–2017)

**DOI:** 10.1038/s41598-019-41950-3

**Published:** 2019-04-02

**Authors:** Mariana Sequetin Cunha, Antonio Charlys da Costa, Natália Coelho Couto de Azevedo Fernandes, Juliana Mariotti Guerra, Fabiana Cristina Pereira dos Santos, Juliana Silva Nogueira, Leandro Guariglia D’Agostino, Shirley Vasconcelos Komninakis, Steven S. Witkin, Rodrigo Albergaria Ressio, Adriana Yurika Maeda, Fernanda Gisele Silva Vasami, Ursula Mitsue Abreu Kaigawa, Laís Sampaio de Azevedo, Paloma Alana de Souza Facioli, Fernando Luiz Lima Macedo, Ester Cerdeira Sabino, Élcio Leal, Renato Pereira de Souza

**Affiliations:** 1 Vector-Borne Diseases Group, Virology Center, Adolfo Lutz Institute, Sao Paulo, Brazil; 2 Institute of Tropical Medicine, University of Sao Paulo, Sao Paulo, Brazil; 3 Pathology Center, Adolfo Lutz Institute, Sao Paulo, Brazil; 4 Laboratory of Retrovirology, Federal University of Sao Paulo, Sao Paulo, Brazil; 5 Post-Graduate Program in Health Sciences, FMABC, Santo Andre, Brazil; 6 Department of Obstetrics and Gynecology, Weill Cornell Medicine, New York, New York USA; 7 Institute of Biological Sciences, Federal University of Para, Belem, Brazil

## Abstract

Beginning in late 2016 Brazil faced the worst outbreak of Yellow Fever in recent decades, mainly located in southeastern rural regions of the country. In the present study we characterize the Yellow Fever Virus (YFV) associated with this outbreak in São Paulo State, Brazil. Blood or tissues collected from 430 dead monkeys and 1030 pools containing a total of 5,518 mosquitoes were tested for YFV by quantitative RT-PCR, immunohistochemistry (IHC) and indirect immunofluorescence. A total of 67 monkeys were YFV-positive and 3 pools yielded YFV following culture in a C6/36 cell line. Analysis of five nearly full length genomes of YFV from collected samples was consistent with evidence that the virus associated with the São Paulo outbreak originated in Minas Gerais. The phylogenetic analysis also showed that strains involved in the 2016–2017 outbreak in distinct Brazilian states (i.e., Minas Gerais, Rio de Janeiro, Espirito Santo) intermingled in maximum-likelihood and Bayesian trees. Conversely, the strains detected in São Paulo formed a monophyletic cluster, suggesting that they were local-adapted. The finding of YFV by RT-PCR in five *Callithrix* monkeys who were all YFV-negative by histopathology or immunohistochemistry suggests that this YFV lineage circulating in Sao Paulo is associated with different outcomes in *Callithrix* when compared to other monkeys.

## Introduction

Yellow fever virus (YFV), a member of the *Flavivirus genus*, is the prototype virus of the *Flaviviridae* family. It is transmitted to susceptible hosts (humans or non-human primates) by the bite of infected mosquitoes, and is considered endemic in parts of Africa and South America, including Brazil. Due to the variety of clinical symptoms elicited in YFV-infected individuals, as well as the presence of asymptomatic infections, the true incidence of YFV infection is believed to be 10 to 50-fold higher than the official reports^1^.

Historically, two YFV transmission cycles occur in Brazil: the sylvatic and the urban cycles. The sylvatic cycle involves *Haemagogus* sp. and *Sabethes* sp. mosquito vectors and several species of non-human primates (NHP) as hosts, mainly *Alouatta* sp., *Sapajus* sp., and *Callithrix* sp.^2^. Most New World NHP, especially those of the *Alouatta* genus, are susceptible to YFV. The Brazilian surveillance system is based upon YFV detection in dead monkeys. Its presence triggers mass vaccination of the local population in response to potential YFV epizootics^2^. In urban areas, however, YFV is transmitted by *Aedes aegypti* mosquitoes. The urban cycle was considered eradicated in 1942 in Brazil following years of vector control programs and vaccination.

During the late 20th century and throughout the first decade of the 21st century, YFV circulation extended from the Amazon region to the adjoining states of Goias and Mato Grosso do Sul (Central Brazil)^3^. Phylogenetic analysis demonstrated that two YFV genotypes were present in South America (SA): SA genotype I, which included strains recovered from Brazil, Panama, Colombia, Ecuador, Venezuela, and Trinidad, and SA genotype II, which included viruses recovered mainly from Peru^4,5^. The Brazilian strains have been divided into two major subclades with four clusters (1A-1D) within clade SA I, showing a complex pattern of geographic and temporal associations^6^. In 2008 and 2009, a new YFV spread was observed in Brazil, with the virus reaching the southern and the southeastern regions of the country^7^. This was outside the recognized YFV endemic/enzootic area and there had been no prior anti-YFV vaccine recommendations. It was demonstrated that a new subclade 1E within the clade 1 of the South American genotype was responsible for the epizootic/epidemic events in the State of São Paulo^8^. Between March and April 2009 this outbreak accounted for a total of 28 human cases with 11 deaths and 99 epizootic events in animals distributed in 36 different counties^9^.

More recently, starting in late 2016 until May 2017, Brazil experienced the worst outbreak of Yellow Fever in the past decades, with 792 confirmed human cases and 274 deaths, mainly located in the southeastern region of the country (Espirito Santo 260 cases, Minas Gerais 487 cases, Rio de Janeiro 17 cases and São Paulo 20 cases). (http://portalarquivos.saude.gov.br/images/pdf/2017/junho/02/COES-FEBRE-AMARELA---INFORME-43---Atualiza----o-em-31maio2017.pdf) ^10^.

In the present study we describe features of YFV strains isolated between 2016 and 2017 during the epizootic events in São Paulo State.

## Materials and Methods

### Study area

The study was conducted in the state of São Paulo, Brazil, which is composed of 645 cities divided into 15 administrative regions, and occupies an area of approximately 248,1796,960 square km with 44,749,699 inhabitants, concentrated mainly in the coastal zone.

### YFV detection in non-human primates (NHPs)

Tissues from dead monkeys collected by local authorities were sent to the local reference laboratory (Adolfo Lutz Institute, São Paulo) for YFV detection. Fresh tissues from 430 monkeys were used for molecular detection of the viral genome, while tissues fixed in 10% neutral buffered formalin were used for routine histopathology with hematoxylin and eosin (H&E) and immunohistochemistry (IHC). Viral RNA was extracted using the QIAamp RNA Blood Mini Kit (QIAGEN, Hilden, Germany), following manufacturer’s instructions. Amplification of the YFV genome employed the TaqMan real-time RT-quantitative PCR (RT-qPCR) protocol^11^, which targets the highly conserved 5′noncoding region (5′NC). Liver and brain were used for RT-qPCR diagnosis. However, when these tissues weren’t available, spleen or lung were substituted. If NHP tissues were not sent to the laboratory, whole blood was used for YFV diagnosis by viral isolation in C6/36 cell lines followed by indirect immunofluorescent assay (IFA)^12^.

For IHC analysis, liver tissue sections were tested with an *in house* primary polyclonal anti-YFV antibody (1:40.000 dilution). Signal amplification was achieved with Super HighDef™ (Enzo Life Sciences, Farmingdale, USA) and visualization was done with the use of diaminobenzidine (D-5637; Sigma, St. Louis, MO, USA).

### Mosquito collection

A total of 5,518 mosquitoes were collected at sites of ongoing epizootic events and in adjacent cities during the study period. They were identified by Sucen (*Superintendence for Control of Endemic Diseases*, *State of São Paulo*) and separated according to species, place and date of collection into 1,030 pools containing 1–50 mosquitoes per pool. (Table 1). Pools were triturated in sterile grinders containing 1 mL of phosphate-buffered saline solution with 0.75% bovine albumin, penicillin (100 units/mL) and streptomycin (100 µg/mL). The resultant suspension was centrifuged at 1800 × g for 15 min. The supernatant was withdrawn and frozen at −70 °C.

**Table 1:** Total number of pools inoculated into the C6/36 cell line from 66 cities in São Paulo State, between October 2010 and March 2017.

Culicid	Number of pools	%
*Aedes aegypti*	63	6.1
*Aedes albopictus*	253	24.6
*Aedes argyrothorax*	8	0.8
*Aedes purpureus*	1	0.1
*Aedes scapularis*	239	23.2
*Aedes serratus*	49	4.8
*Aedes spp*	3	0.3
*Aedes terrens aff*.	9	0.9
*Haemagogus janthinomys*	1	0.1
*Haemagogus janthinomys/capricornii*	7	0.7
*Haemagogus leucocelaenus*	71	6.9
*Psorophora (Jan.) sp*	4	0.4
*Psorophora albigenu*	2	0.2
*Psorophora albipes*	10	1.0
*Psorophora ferox*	133	12.9
*Psorophora lanei*	1	0.1
*Psorophora varipes/albigenus*	7	0.7
*Psorophora spp*	2	0.2
*Sabethes albiprivus*	1	0.1
*Sabethes belisarioi (aff)*	10	1.0
*Sabethes chloropterus*	18	1.8
*Sabethes glaucodaemon*	50	4.9
*Sabethes gymnothorax*	1	0.1
*Sabethes imperfectus*	4	0.4
*Sabethes intermedius*	11	1.1
*Sabethes petrocchiae*	5	0.5
*Sabethes purpureus*	49	4.8
*Sabethes shannoni*	1	0.1
*Sabethes tridentatus*	2	0.2
*Sabethes undosus aff*.	14	1.4
*Sabethes sp*	1	0.1

### YFV isolation

For virus isolation, 20 µL of supernatant from each mosquito pool was inoculated into cell tubes containing monolayer cultures of C6/36 cells. Liver, brain or spleen samples from 59 positive RT-qPCR NHPs were manually macerated and diluted in L-15 medium. When available previously diluted whole blood was similarly inoculated. C6/36 culture tubes were incubated for nine days at 28 °C with L-15 medium containing 2% FBS, penicillin (100units/mL) and streptomycin (100 µg/mL). IFA tests were performed using YFV monoclonal antibodies provided by the U.S. Centers for Disease Control and Prevention.

### Sequencing

Whole genomes were obtained from positive NHP and mosquito pools inoculated into C6/36 cell culture specimens after one passage. The specimens were clarified by centrifugation at 12,000 × g for ten minutes and then filtered through a 0.45-µm filter (Millipore). The filtrates were treated with a mixture of nuclease enzymes to digest unprotected nucleic acids. Viral nucleic acids were extracted using a Maxwell 16 automated extractor (Promega). Viral cDNA synthesis from extracted viral RNA/DNA using 50 pmol of an octamer of random primer in a reverse transcription reaction with SuperScript III (Life Tech) was performed. The 2nd strand cDNA synthesis was performed using DNA Polymerase I Large (Klenow) Fragment (Promega), followed by the use of a Nextera XT Sample Preparation Kit (Illumina) to construct a DNA library with each sample identifiable using dual barcodes. For size selection, we used a Pippin Prep (Sage Science, Inc) to select a 400 bp insert (range 200–600 bp). The library was deep-sequenced using the MiSeq Illumina platform with 300 bp paired ends. Datasets were then trimmed according to the quality (99.9% coverage) and length (reads < 30 bp were removed) of each read using Geneious R9 software (Biomatters Ltd L2, 18 Shortland Street Auckland, 1010, New Zealand). Near full genome sequences were then reconstituted by mapping the reads to a reference sequence from GenBank. Sequences obtained were examined to ensure that the mapping to a reference sequence did not generate a biased consensus sequence.

### Alignment and phylogenetic analysis

BLASTn was initially used to identify viral sequences through their similarity to annotated viral genomes in GenBank. Based on the best hits of the BLASTn, the following 101 genomes, listed by their Genbank numbers, were chosen to be used in the next analyses: MH666057; MH666060; MH666056; MH666058; MH484426; MH018095; MH484427; MF370532; MF370538; MF370534; MH018096; MF370535; MH018090; MH018080; MH018079; MF423375; MF423374; MF423373; MF538783; MF370549; MF370548; MF370547; MF370537; MF370536; MF370533; MH018078; MF538786; MF538785; MF538784; MF538782; MF423378; MF423377; MF423376; MF434851; MF170973; MH018092; MF370531; MF170972; KY885000; MH484429; MF170977; MF170976; MF370530; MF170979; MF170975; MF170981; MF170974; MF170970; MF170978; MF170969; MH018093; MH018076; MH484434; MH018083; MH018099; MF170980; MH018091; MF170968; MF170971; MH484430; MH018084; MH018089; MH018088; MH018065; MH018066; MH018067; MH018082; MH018064; MF370546; MF370544; MF370540; KM388817; KM388816; KM388814; JF912190;KY861728; MF370541; KM388818; KM388815; MF370543; MF370539; MF370542; JF912188; JF912187; JF912189; MG969501; MH018101; MH018100; HM582851; MF370545; MF347613; JF912180; JF912182; JF912185; JF912179; JF912183; JF912186; JF912184; MH484425; MH018074 and MF185660.

These genomes were then aligned using Clustal X software^13^. Subsequently, a phylogenetic tree was constructed using the Maximum Likelihood approach, and branch support values were assessed using the Shimodaira-Hasegawa test. All trees were inferred using FastTree software^14^. The GTR model and gamma distribution were selected according to the likelihood ratio test (LRT) implemented in the jModeltest software^15^.

### Coalescent analysis

We used a Bayesian Markov chain Monte Carlo (BMCMC) coalescent framework to estimate ancestral genealogy, phylodynamics and evolutionary parameters such as nucleotide substitution rates per year and time to the most common ancestor (tMRCA). The BMCMC method incorporates the uncertainty of the measurements by considering the errors intrinsic in both the tree reconstruction and the coalescent method. The GTR model plus a gamma correction (Γ) was applied to all analyses; the evolutionary and demographic parameters were iteratively adjusted. We used constant population size coalescent prior models to determine the key parameters to study the spatio-temporal spread of YFV in Brazil. In our analyses, sequences sampled at different times (heterochronous) were used to estimate the ancestral genealogy, evolutionary parameters such as nucleotide substitution rates per year and diversity over time. The Bayesian stochastic search variable selection (BSSVS) approach was used to infer the ancestral reconstruction of discrete states (i.e., location) in a Bayesian statistical framework for evolutionary hypothesis testing^16^. BSSVS infers a Bayes factor hypothesis test that identifies the most parsimonious description of the phylogeographic diffusion process in time-frame calibrated under a strict or relaxed molecular clock, allowing characters to be mapped in natural time-frames. The BSSVS was used to estimate posterior probabilities allowing us to perform a Bayes factor test to describe the most parsimonious phylogeographic diffusion process of the recent YFV outbreak in São Paulo. This approach permits the ancestral reconstruction of discrete states (here the geographical location of YFV strains) to be estimated in a Bayesian statistical model for evolutionary hypothesis testing towards the root on real-time calibrated coalescent trees. The estimated posterior probabilities (used for the Bayes factor test) of location mapping in time-calibrated trees under a relaxed molecular clock (in combination with constant population size) can be summarized to show spatial-temporal diffusion process in a geographical framework). We used this full probabilistic approach to study YFV phylogeography and host spread in the time interval between 1980 and 2017. The Markov chain Monte Carlo (MCMC) processes were run for 250,000,000 generations with the initial 10% of each run discarded as burn-in. The convergence of chains was evaluated using TRACER software, version 1.7.1^17^. Runs were accepted when all parameters presented an effective sample size number (ESS) >200. Two independent chains were run for each dataset and combined with LogCombiner software. The TreeAnnonator software was used to summarize parameters in a Maximum Clade Credibility (MCC) tree. All these analyses were performed with the BMCMC approaches implemented in the BEAST package version 1.10.2^18^. We used this summarized MCC tree to visualize the spatial phylogenetic processes of dissemination in geographic space. The software Spread 3D v0.9.7^19^ was used to describe the temporal-spatial diffusion of YFV through time.

### Biosafety

All protocols and procedures were conducted within the enhanced laboratory biosafety level 2 (ABSL-2) facility of Institute Adolfo Lutz. The ABSL-2 facility consists of a laboratory in which all *in vitro* experimental work is carried out in class 3 biosafety cabinets, which are also negatively pressurized (<−200 Pa). Although all experiments were conducted in closed class 3 cabinets and isolators, special personal protective equipment, including laboratory suits, gloves, and FFP3 facemasks were used. Air released from the class 3 units was filtered by High-Efficiency Particulate Air (HEPA) filters and then released via the facility ventilation system, again via HEPA filters. Only authorized personnel who received the appropriate training can access the facility. The facility is secured by procedures recognized as appropriate by the institutional biosafety officers and facility management at São Paulo University and Brazilian National Technical Biosafety Commission

### Ethical statement

All animal research was approved by the Institutional Animal Care and Use Committee (IACUC) of the Adolfo Lutz Institute, São Paulo. The Adolfo Lutz Central Institute (The Central Public Health Laboratory from the State of São Paulo), an organ linked to the Health Department of the state of São Paulo, is the official laboratory for the diagnosis of YFV in humans and primates. NHP samples were sent by local authorities in accordance with Brazilian Ministry of Health guidelines (http://vigilancia.saude.mg.gov.br/index.php/download/guia-de-epizootias-febre-amarela-2a-edicao-2017/#). Previous approval by ethics committees of the Adolfo Lutz Institute and the College of Medicine from the University of São Paulo were granted. This was an anonymous, unlinked study and informed consent was not required according to resolution and the entire protocol was approved by the Consultative Committee for Ethics and Animal Experimentation of the Adolfo Lutz Institute and the College of Medicine from the University of São Paulo, Brazil. The use of NHP samples for research was also approved by ICMBIO protocol number 65181 (http://www.icmbio.gov.br/sisbio/).

## Results

### YFV detection in NHP and mosquitoes from São Paulo State

**Figure 1:**
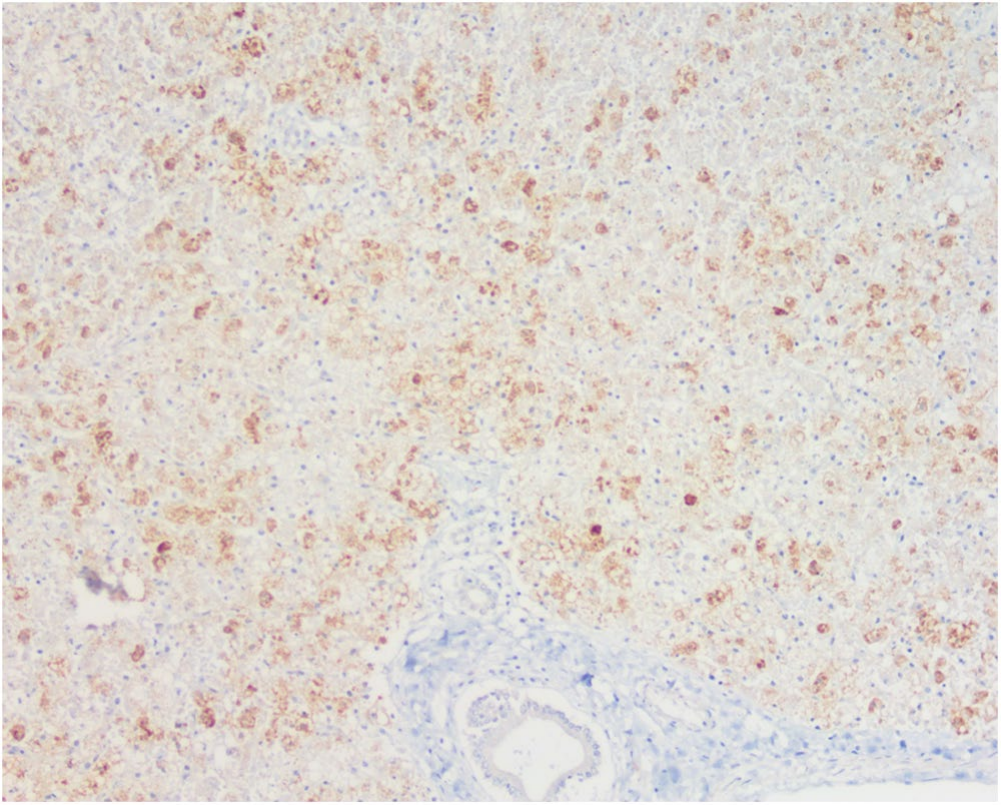
Positive immunolabeling confined to remaining periportal hepatocytes and terminal plate. Original magnification ×40; immunohistochemical staining for yellow fever virus.

**Figure 2:**
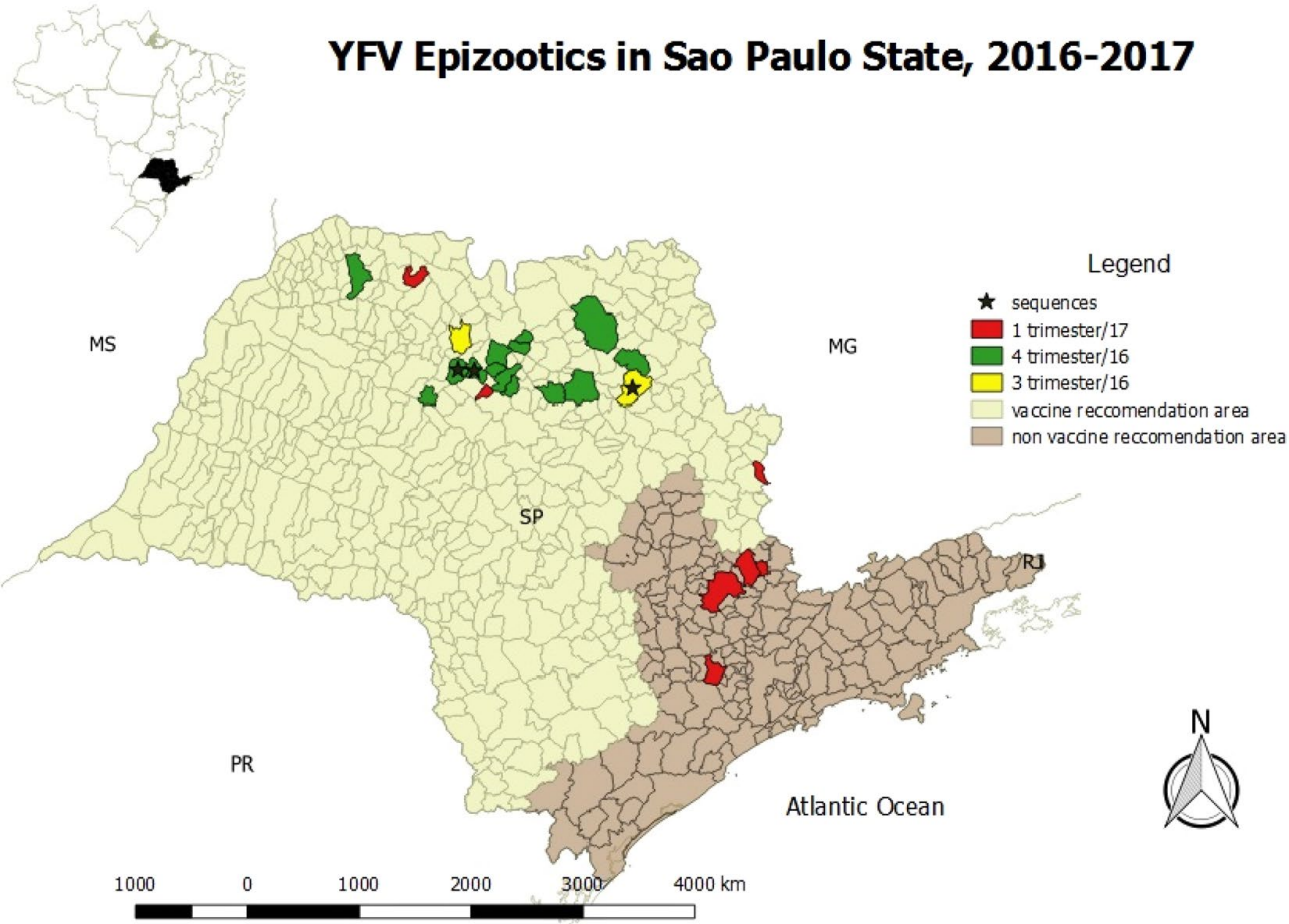
Yellow Fever Virus distribution in non-human primates by trimester (2016–2017) in São. Paulo State. Maps were created using the QGIS software version 3.0.0 (Girona), available in https://qgis.org.

Carcasses of NHPs belonging mainly to the genus *Alouatta* spp. (howler monkey), *Callithrix* spp. (marmosets), and *Sapajus* spp., but also a smaller proportion of *Callicebus nigrifrons* and the endangered species *Leontopithecus rosalia*, were tested. For RT-qPCR, a NHP was considered positive if one tissue sample had a Cq value ≤ 38. For IHC, all samples that showed brown granular cytoplasmic hepatocytes on immunolabeling were considered positive. Figure 1 shows immunohistochemical findings in the livers of neotropical NHPs that died of yellow fever. The first YFV epizootic in São Paulo State was detected by the *Vector-Borne Diseases Group* (Adolfo Lutz Institute) in July 2016 in a *Callithrix spp*. monkey found dead in the center of Ribeirao Preto, a city located in the northwest of São Paulo State, with about 600.000 inhabitants. The second epizootic was detected in August 2016 in an *Alouatta* spp. monkey from Sao Jose do Rio Preto, 200 Km distant from the first confirmed area. Notably, between the end of January 2017 and March 2017, YFV expanded towards the Atlantic coast of Brazil in areas not previously considered at risk for yellow fever, as a new epizootic wave was confirmed in the center of the São Paulo State, the most populous area and closer to São Paulo City. Figure 2 shows the map of São Paulo State with the 25 cities where epizootic events have been detected during the studied time period and its dissemination by trimester, including to areas where vaccination had not been recommended. Black asterisks show cities from where data were used for the phylogenetic analysis, as detailed below.

**Figure 3:**
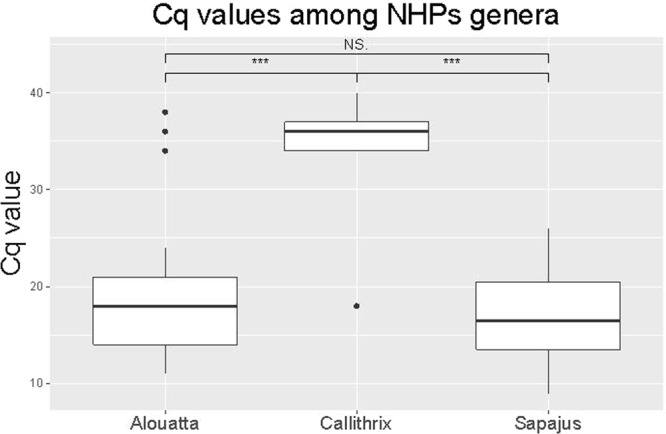
Boxplot of quantification cycles (Cq) values distribution of yellow fever virus between non-human primate genera. The thick horizontal line indicates the median. Gray boxes and vertical lines indicate interquartile range and the variance between Cq values per genera, respectively. Statistical analysis (analysis of variance) shows a significant difference between *Alouatta* and *Callithrix* (p < 0.000) and *Sapajus* and *Callithrix* (p < 0.000). There were no statistical differences between *Alouatta* and *Sapajus* (p = 0.302). Ns: not significant, ^***^p-value < 0.01.

During the study period, 67 NHPs were positive for YFV by IHC or RT-qPCR, and one NHP was confirmed positive by IFA. From these 67 NHPs, 30 were *Alouatta* spp., nine *Sapajus* spp., seven *Callithrix* spp, and 21 animals were not identified by genus. A total of 367 animals were tested by RT-qPCR and IHC, and two animals were tested only by IFA. Table 2 shows positive animals by RT-qPCR, IHC and IFA, location and whether the epizootic event occurred in an area previously free of vaccination. When comparing the different diagnostic methods, 9 animals that were tested by both RT-qPCR and IHC were positive only by gene amplification. Samples from three of these animals presented with autolysis (animal ID 17, 22 and 30), five samples belonged to the genus *Callithrix* spp. (animal ID 1, 6, 20, 29 and 36) and one belonged to the genus *Alouatta* spp. (animal ID 4). None of these monkeys had signs of yellow fever after analysis of liver sections by H&E staining. Regarding the molecular detection of YFV using RT-qPCR, Cq values ranged from 18 to 38, with an average of 30.33 in *Callithrix* spp., from 11 to 38, with an average of 16.3 in *Alouatta* spp. and from 11 to 26, with an average of 17 in *Sapajus* spp. Figure 3 shows a boxplot with Cq differences between NHP genera, suggesting that *Callithrix* monkeys had lower viral loads than the other two genera (p < 0.005).

**Table 2:** Yellow Fever positive NHP in São Paulo State between July 2016 and March 2017 by RT-qPCR, IHC or IFA.

Animal ID	Genera/Species	City	Mesoregion	Vaccine Recomendation	RT-qPCR	Tissue	IHC
1	*Callithrix spp*.	Ribeirao Preto	Ribeirao Preto	Y	P	P in liver/N in brain	N
2	*Sapajus spp*.	Potirendaba	Sao Jose do Rio Preto	Y	P	P in liver and brain	P
3	*Alouatta spp*.	Pindorama	Sao Jose do Rio Preto	Y	P	P in liver and brain	P
4	*Alouatta spp*.	Ibirá	Sao Jose do Rio Preto	Y	P	P in brain/N in liver	N
5	*Callithrix spp*.	Ribeirao Preto	Ribeirao Preto	Y	P	N in liver/P in brain	NP
6	*Callithrix spp*.	Ribeirao Preto	Ribeirao Preto	Y	P	P in liver and brain	N
7	*Sapajus spp*.	Cajobi	Sao Jose do Rio Preto	Y	P	P in liver and brain	NP
8	*Alouatta spp*.	Adolfo	Sao Jose do Rio Preto	Y	P	P in liver and brain	P
9	*Sapajus spp*.	Pindorama/SP	Sao Jose do Rio Preto	Y	P	P in liver and brain	P
10	*Sapajus spp*.	Pindorama/SP	Sao Jose do Rio Preto	Y	P	P in liver and brain	P
11	*Alouatta spp*.	Catanduva	Sao Jose do Rio Preto	Y	P	P in liver and brain	P
12	*Alouatta spp*.	Catanduva	Sao Jose do Rio Preto	Y	P	P in liver and brain	NP
13	*Alouatta spp*.	Catiguá	Sao Jose do Rio Preto	Y	P	P in liver and brain	P
14	*Alouatta caraya*	Jaboticabal	Ribeirao Preto	Y	P	P in liver and brain	NP
15	*Alouatta caraya*	Jaboticabal	Ribeirao Preto	Y	P	P in liver and brain	NP
16	*Alouatta caraya*	Jaboticabal	Ribeirao Preto	Y	P	P in liver and brain	NP
17	*Alouatta spp*.	Morro Agudo	Ribeirao Preto	Y	P	P in liver and brain	A
18	*Alouatta spp*.	Severínia/SP	Sao Jose do Rio Preto	Y	P	P in liver and brain	P
19	*Alouatta caraya*	Jaboticabal	Ribeirao Preto	Y	P	P in liver and brain	P
20	*Callithrix penicillata*	Jardinópolis	Ribeirao Preto	Y	P	P in liver and brain	N
21	*Alouatta caraya*	Jaboticabal	Ribeirao Preto	Y	P	P in liver and brain	P
22	*Callithrix spp*.	Fernandópolis	Sao Jose do Rio Preto	Y	P	N in liver/P in brain	A
23	*Alouatta spp*.	Catiguá	Sao Jose do Rio Preto	Y	P	P in liver and brain	P
24	*Sapajus spp*.	Tabapua	Sao Jose do Rio Preto	Y	NP	NP	NP
25	*Sapajus spp*.	Marapoama	Sao Jose do Rio Preto	Y	P	P in liver and brain	P
26	*Alouatta spp*.	Ribeirao Preto	Ribeirao Preto	Y	P	P in liver and brain	P
27	*Alouatta spp*.	Jaboticabal	Ribeirao Preto	Y	P	P in liver and brain	P
28	*Alouatta caraya*	Ribeirao Preto	Ribeirao Preto	Y	P	P in liver and brain	P
29	*Callithrix spp*.	Sao Jose do Rio Preto	Sao Jose do Rio Preto	Y	P	P in brain/N in liver	N
30	*Alouatta spp*.	Americo de Campos	Sao Jose do Rio Preto	Y	P	P in brain and spleen	A
31	*Alouatta spp*.	Sao Roque	São Paulo	No	P	P in liver/N in spleen	NP
32	*Sapajus spp*.	Águas da Prata	Campinas	No	P	P in brain and spleen	P
33	*Sapajus spp*.	Águas da Prata	Campinas	No	P	P in brain and spleen	P
34	*Alouatta spp*.	Amparo	Campinas	No	P	P in brain and spleen	P
35	*Alouatta spp*.	Monte Alegre do Sul	Campinas	No	P	P in brain and spleen	P
36	*Callithrix spp*.	Fernandopólis	Sao Jose do Rio Preto	Y	P	P in liver and brain	N
37	*NI*	Amparo	Campinas	No	P	P in liver and spleen	P
38	*NI*	Amparo	Campinas	No	P	P in liver and spleen	P
39	*NI*	Amparo	Campinas	No	P	P in liver and spleen	P
40	*NI*	Monte Alegre do Sul	Campinas	No	P	P in liver and spleen	P
41	*Sapajus spp*.	Socorro	Campinas	No	P	P in liver and spleen	P
42	*Alouatta caraya*	Campinas	Campinas	No	P	P in liver and spleen	P
43	*Alouatta caraya*	Campinas	Campinas	No	P	P in liver and spleen	P
44	*Alouatta caraya*	Campinas	Campinas	No	P	P in liver and spleen	P
45	*NI*	Monte Alegre do Sul	Campinas	No	P	P in liver and spleen	P
46	*NI*	Monte Alegre do Sul	Campinas	No	P	P in liver and spleen	P
47	*NI*	Amparo	Campinas	No	P	P in liver and spleen	P
48	*NI*	Amparo	Campinas	No	P	P in liver and spleen	P
49	*NI*	Monte Alegre do Sul	Campinas	No	P	P in liver and spleen	P
50	*NI*	Monte Alegre do Sul	Campinas	No	P	P in liver and spleen	P
51	*NI*	Tuiuti	São Paulo	No	P	P in liver and spleen	P
52	*NI*	Monte Alegre do Sul	Campinas	No	P	P in liver and spleen	P
53	*NI*	Monte Alegre do Sul	Campinas	No	P	P in liver and spleen	P
54	*NI*	Monte Alegre do Sul	Campinas	No	P	P in liver and spleen	P
55	*NI*	Amparo	Campinas	No	P	P in liver and spleen	P
56	*Alouatta fusca clamitans*	Pinhalzinho	Campinas	No	P	P in liver and spleen	P
57	*Alouatta spp*	Campinas	Campinas	No	P	P in liver and spleen	P
58	*NI*	Monte Alegre do Sul	Campinas	No	P	P in liver and spleen	P
59	*NI*	Monte Alegre do Sul	Campinas	No	P	P in liver and spleen	P
60	*NI*	Monte Alegre do Sul	Campinas	No	P	P in liver and spleen	P
61	*Alouatta spp*	Campinas	Campinas	No	P	P in liver and spleen	P
62	*NI*	Monte Alegre do Sul	Campinas	No	P	P in liver and spleen	P
63	*NI*	Monte Alegre do Sul	Campinas	No	P	P in liver and spleen	P
64	*NI*	Monte Alegre do Sul	Campinas	No	P	P in liver and spleen	P
65	*Alouatta spp*.	Campinas	Campinas	No	P	P in liver and spleen	P
66	*Alouatta spp*.	Campinas	Campinas	No	P	P in liver and spleen	P
67	*Alouatta spp*.	Campinas	Campinas	No	P	P in liver and spleen	P

NI: not informed, P: positive, N: negative, NP: not performed, A: autolyzed, Y: yes.

From 1030 culicid pools inoculated into C6/36 cell lines only 3 were positive for YFV (0.29%) after one passage. Two of these pools were obtained from *Haemagogus janthinomys* and one from *Haemagogus leucocelaenus*, all from Ribeirao Preto city. After 3 passages in C6/36 cells, YFV was isolated from 22 NHP samples belonging only to *Alouatta* and *Sapajus* genera. We could not isolate YFV from *Callithrix* monkeys, probably due to low viral loads in this genus. Whole genomes were obtained from 3 viral isolates obtained from NHPs, collected in Ribeirao Preto (*Alouatta caraya* accession number MH666057), Catanduva (*Alouatta* spp. accession number MH666056) and Tabapua (*Sapajus* spp. accession number MH666058) and from 2 isolates obtained from mosquito pools (*Haemagogus leucocelaenus* accession number MH666059 and *Haemagogus janthynomis/capricornii* accession number MH666060).

### Phylogenetic Analysis of YFV strains in South America

**Figure 4:**
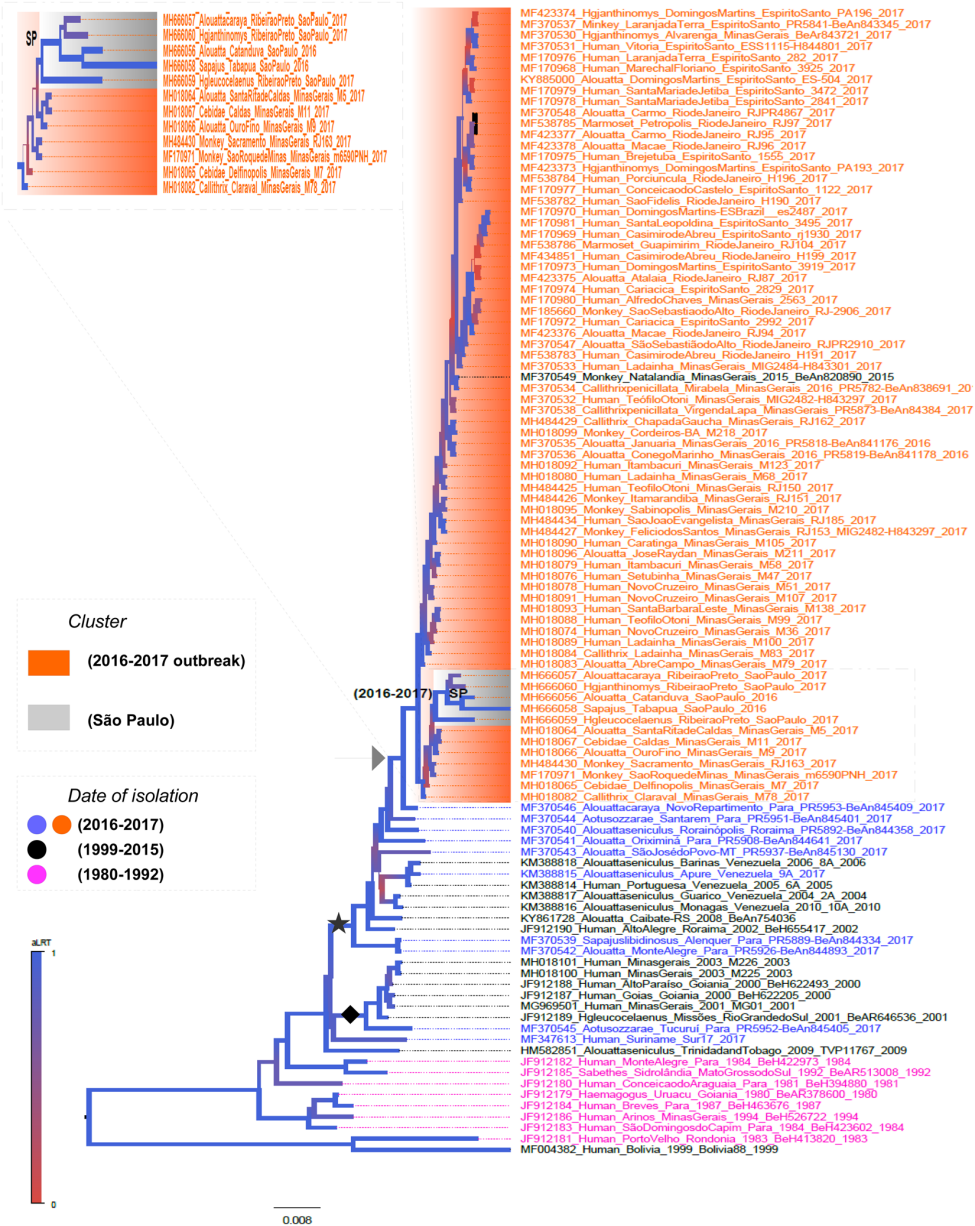
Maximum likelihood tree of yellow fever virus (YFV). Strains from the 2016–2017 outbreak are highlighted in orange in the tree. Isolates sequenced in this study are indicated by gray. The tree was constructed using the Maximum Likelihood approach, and branch support values were assessed using the Shimodaira-Hasegawa test. All trees were inferred using FastTree software^14^. The GTR model and gamma distribution were selected according to the likelihood ratio test (LRT).

**Figure 5:**
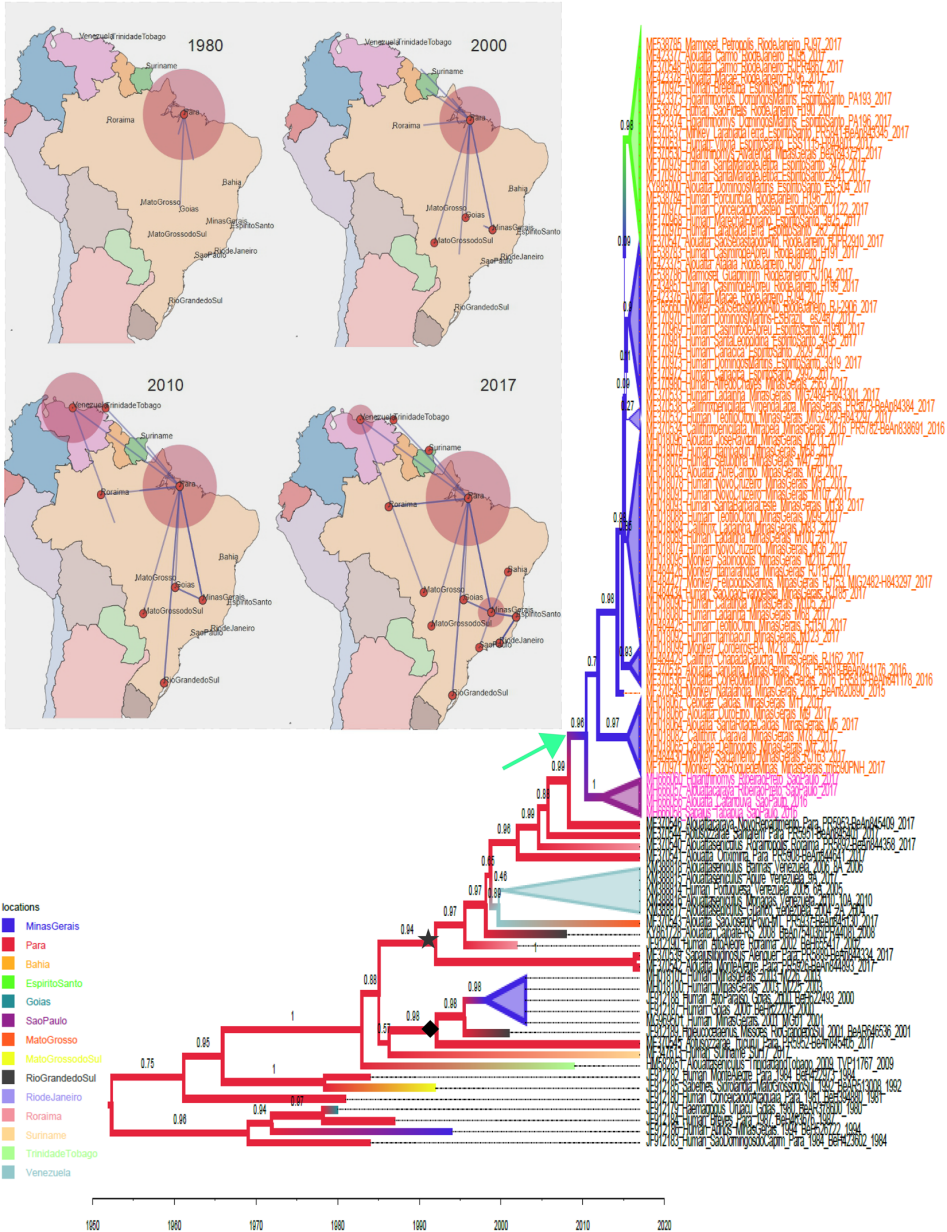
Geographical dissemination of yellow fever virus (YFV) between 1980 and 2017. Temporal stages of the phylogeographic spread of YFV are shown in each panel. Lines indicate the dissemination route of YFV and are based on the location probability of each node in the MCC tree (Bayes-factor > 2). The size of the circular areas shown in different location regions is proportional to the number of lineages in the MCC tree at a certain time interval. Countries and Brazilian states from where YFV lineages were included in the analysis are named in the map. YFV isolates from Brazil, Venezuela, Suriname, and Trinidad & Tobago were analyzed. Samples from Brazil were sampled between 1980 and 2017. The MCC tree used to summarize the phylogeographic process is also shown. Branches were colored according to the most probable location in the tree. The location color code is indicated in the lower left panel. Values at each node in the tree indicate the posterior probability of certain location. Some clades were collapsed in order to facilitate the visualization of the tree. The x-axis represents the chronological time, expressed in years. All Bayesian analyses were performed using the software Beast version 1.10 and the MCC tree was summarized using the TreeAnnonator software. The MCC tree was used to construct time-frame maps showing the temporal-spatial diffusion of YFV. The dispension of YFV through time was inferred using software Spread 3D v0.9.7.

We used nearly all full-length genomes of YFV (n = 101) available in Genbank to assess the relatedness of our sequences. The topology of the maximum likelihood (ML) tree showed that YFV strains of genotype I have a temporal pattern; historic sequences from 1980 to 1990 (indicated in magenta) are located at the base, sequences from 1990 to 2015 (indicated in black) are intermediate and sequences from 2016–2017 outbreak (indicated in orange in the tree) are located in the tips (Fig. 4). We used sequences JF912181 and MF004381 of YFV genotype II as outgroup (Fig. 4). The ML tree also showed that YFV from São Paulo (indicated by the gray area in the tree) grouped within the cluster of sequences associated with the 2016–2017 YFV outbreak (indicated by the orange area in the tree). YFV strains at the base of this cluster (indicated by the black star) are from Venezuela and mainly from states in the north region of Brazil (i.e., Para and Roraima). This is in agreement with a previous study suggesting that the 2016–2017 YFV outbreak disseminated from the north and spread towards southeast regions of Brazil^20^. The ML tree also showed that there are two YFV lineages currently circulating in the north region of Brazil; one that gave rise to the 2016–2017 outbreak (indicated by a black star in the tree) and the outgroup is strain BeH655417 from 2002 in north Brazil. Another lineage (indicated by black dot) is that of a previous YFV outbreak in 2000–2003 in Minas Gerais (southeast Brazil); it contains YFV sequences from the central regions and the south state of Rio Grande do Sul. The outgroup is BeAn845405 isolated in 2017 in north Brazil. The tree topology that showed two lineages of YFV of South American isolates of genotype I was further confirmed by Bayesian inferences (indicated by a black star and a black diamond in the tree in Fig. 5).

### Dissemination of YFV 2016–2017 outbreak

Besides the relatedness of lineages, we also used the associated information regarding the location where each virus was isolated. The Bayesian stochastic search variable selection (BSSVS) approach and symmetric substitution model were used to infer the ancestral reconstruction of location as a discrete state to be mapped in the internal nodes of an MCC tree (Fig. 5). The summarized maximum clade credibility (MCC) tree shows the posterior probabilities (PP) of each location indicated by values and colors of branches (see the location color code in the panel of Fig. 5). Sequences related with the 2016–2016 outbreak are indicated in orange in the tree and sequences from São Paulo in magenta. The MCC tree also shows that Minas Gerais (southeast Brazil) is the most likely place from where YFV reached São Paulo. The ancestral node shared by all strains related to the 2016–2017 YFV outbreak (orange area in the tree) has a high posterior probability (PP = 0.96, indicated by green arrow) associated with Minas Gerais (indicated in blue in the MCC tree). The MCC was used to show the phylogenetic diffusion of YFV in a geographic time-space. The results are shown in Fig. 5, presenting static time frames of YFV dissemination. In 1980, the virus was endemic in the Brazilian state of Para (the size of red circle indicate the number of isolates in the 1980s), and from this region YFV began to spread to other central regions of the country. The dissemination routes are indicated by blue lines. By the year 2000, YFV reached the states of Mato Grosso do Sul, Goias and Minas Gerais (indicated by red dots), and continued to spread to the Central region of Brazil. The scenario of YFV infection by 2010 indicated that the virus was present in Roraima (Northern region) and one strain was found in Rio Grande do Sul in the extreme Southern region of Brazil. The virus also reached Venezuela and Trinidad and Tobago. YFV reached São Paulo in 2013 and was fully disseminated in most Brazilian states and in Suriname by 2017.

## Discussion

Our study describes the first epizootic events caused by YFV in São Paulo, the most populous state in Brazil, located in the southeastern region of the country. These events began in July 2016 in a previous endemic area, and disseminated to a yellow fever-free area within a few months, confirmed either by RT-qPCR and/or IHC and IFA. Epizootic cases of YFV in the state of São Paulo were confirmed before those reported in Minas Gerais State, where 465 human cases of yellow fever were confirmed between December 2016 and July 2017^21^, and those from Espirito Santo State^22^, where 252 human cases were confirmed. There were 22 cases confirmed in São Paulo during this same period^21^. Differences in the number of human cases was probably due to the silent circulation of YFV or lack of surveillance based on NHPs in Minas Gerais, and highlights the importance of such surveillance activities to prevent human cases through initiation of vaccination programs.

After the YFV epizootics in the northwestern portion of São Paulo State during the second half of 2016, YFV was detected in early 2017 in Campinas, one of the most populous cities in the state of São Paulo, with one million inhabitants. Yellow Fever had not been detected in this city since the beginning of the 20th century when outbreaks of urban yellow fever occurred^7^. The detection of positive NHPs in the Sousas district, in Campinas city, within a 10 Km distance to an important urban center, poses a serious risk for the reintroduction of urban yellow fever, due to a high number of vaccination-naive individuals in the population and the presence of the vector *Aedes aegypti*.

By comparing samples from NHPs by molecular diagnosis and IHC, we suggest that some species belonging to the genus *Callithrix* may have a different susceptibility to YFV when compared to *Alouatta* spp., since 5 non-autolyzed samples from *Callithrix* were positive by RT-qPCR, but had no signs of YFV in liver histology analyses, and negative results in IHC examinations. Nevertheless, we cannot infer the causes of this possible resistance. Previous studies^23^ showed that only a few Brazilian marmosets infected with YFV had hyperthermia, and none had lesions typical of yellow fever as seen in certain other species of monkeys. However, this study from the 1930s used only *Callithrix albicollis* as an animal model^23^. Human and NHP phylogenetic analyses from the current YFV outbreak detected in Rio de Janeiro, Minas Gerais, and Espirito Santo^24–26^ show that the ongoing epizootic events were caused by the SA I genotype. As all YFV cases in NHPs from the southeastern region were caused by the same lineage, we propose that the differences in susceptibility between NHPs are probably host-specific rather than differential YFV virulence. However, the role of *Callithrix* monkeys in the YFV cycle must be further investigated with newer experimental tests, since more than one species within this genus was involved in epizootic events in São Paulo State, including *Callithrix penicillata*, *C*. *jacchus*, and *C*. *aurita*. The presence of hybrid marmosets may also influence the physiopathogenesis of YFV in the *Callithrix* genus. Conversely, the only uncorrelated *Alouatta* result in this study (except for the samples that were autolyzed) was found to be RT-qPCR positive only in brain samples (Cq = 30), but negative in liver samples, probably due to degradation of viral RNA. As IHC is performed only in the liver, this could explain this difference. In addition, the present work detected YFV in 9 *Sapajus* spp. monkeys, by both RT-qPCR and IHC methods. This species was considered less sensitive to the disease, with a low fatality rate^27^. Other studies from previous epizootic events failed to detect YFV in *Sapajus* spp. monkeys^9,28^. On the other hand, NHPs belonging to the *Alouatta* genus were highly infected with YFV as previously reported in this country^29,30^. In fact, as they have high fatality rates and develop a fatal YFV infection similar to those reported in humans, they are the best indicators of YFV circulation.

A variety of New World mosquitoes participate in the sylvatic YFV transmission cycle, including *Haemagogus albomaculatus, Hg. spegazzini, Hg. janthinomys, Sabethes chloropterus, Sa. albiprivus, Sa. glaucodaemon, Sa. soperi* and *Sa. cyaneus*. YFV was also isolated from Psorophora ferox and Aedes serratus in Brazil^2,3^. *Hg leucocelaenus* is likely the main vector in the southeastern region of Brazil, including São Paulo State, although very little data is available concerning viral detection in this region^30–32^. Even though we could not identify other mosquitoes infected with YFV, this active surveillance must be continued to better understand the role of other culicids in the YFV cycle in São Paulo. This is especially relevant in the new areas where several pools of *Aedes albopictus* and *Aedes scapularis* were found, since it has been demonstrated elsewhere that Brazilian *Aedes populations* have the potential of transmitting YFV^33,34^.

Previous reports from the mid-20th century show that other YFV epizootics have begun in northern and western Amazonian regions around 1908 and spread to the South and Central regions of Brazil^35,36^, and later between 1940 and 1990 from Northern Brazil to other Brazilian regions (Central-Western, Northeastern and Southeastern)^36^. These results are in accordance with those shown here, as we demonstrated using complete YFV sequences viral dissemination from Para State, in the Northern region of Brazil, into other Latin American countries and into other Brazilian states in the 2000s. The virus reemerged in São Paulo in 2008–2009, when a YFV lineage 1E was isolated from NHPs in the northeast portion of the State^8^. However, due to the lack of complete genomes from this period, we could not demonstrate how the virus has disseminated during this outbreak. Apparently, this lineage did not circulate after that time in Brazil^36^. YFV did not reach the most populous part of the State as described in 2016–2017, and only *Alouatta* monkeys were found positive at that time using conventional RT-PCR, IHC and virus isolation for diagnosis^9^. This was probably due to the very recent implementation of RT-qPCR^11^ by Brazilian reference laboratories for YFV diagnosis, which is more sensitive than RT-PCR and virus isolation. Moreover, as previously reported, this outbreak was caused by a South American I genotype. This had a probable Venezuelan origin from the epizootic events reported in the 2000s^25,36^, and was introduced into the Southeastern region from an endemic area, possibly northern or center-west Brazil^20^. Despite the fact that the Brazilian Amazon is the major YFV source in South America, it was already demonstrated that there is an independent YFV evolution in Venezuela, which can also disseminate to other Latin American countries^37^. Indeed our phylogenetic analysis based on nearly full length genomes indicated there are two distinct lineages of YFV (genotype I) circulating in the north region of Brazil. The analysis also indicated that YFV strains detected in Sao Paulo are not intermingled with strains from bordering states (Minas Gerais, Rio de Janeiro, Espirito Santo) and instead form a unique phylogroup. This might indicate that YFV strains found in São Paulo are locally adapted and probably migrated from Minas Gerais before 2015.

## Conclusions

The present investigation describes epizootic events caused by South American genotype I of YFV in the state of São Paulo during the 2016–2017 outbreaks when the virus spread to new areas where vaccination had not been previously recommended. We compare the detection of YFV in different NHP genera, showing differences between them. As most differences were noted in *Callithrix* monkeys, additional studies are needed to evaluate YFV infection in this genus and its role in the YFV cycle. This study also reiterates the endemic nature of YFV in Brazil, and emphasizes the need for consistent YFV surveillance and routine mass vaccination of at-risk populations to prevent future outbreaks.
